# A comprehensive prognostic and immune analysis of enhancer RNA identifies IGFBP7-AS1 as a novel prognostic biomarker in Uterine Corpus Endometrial Carcinoma

**DOI:** 10.1186/s12575-022-00172-0

**Published:** 2022-07-15

**Authors:** Jinhui Liu, Jian Yin, Yuanyuan Wang, Lixin Cai, Rui Geng, Mulong Du, Zihang Zhong, Senmiao Ni, Xiaohao Huang, Hao Yu, Jianling Bai

**Affiliations:** 1 Department of Gynecology, The First Affiliated Hospital of Nanjing Medical University, Nanjing, 210029 Jiangsu China; 2 Department of Biostatistics, School of Public Heath, Nanjing Medical University, 101 Longmian Avenue, Jiangning District, Nanjing, 211166 P.R. China

**Keywords:** IGFBP7-AS1, IGFBP7, Enhancer RNA, UCEC, Tumor immunity

## Abstract

**Background:**

Long non-coding RNAs (lncRNA) have been implicated in a hand of studies that supported an involvement and co-operation in Uterine Corpus Endometrial Carcinoma (UCEC). Enhancer RNAs (eRNA), a functional subtype of lncRNA, have a key role throughout the genome to guide protein production, thus potentially associated with diseases.

**Methods:**

In this study, we mainly applied the Cancer Genome Atlas (TCGA) dataset to systematically discover crucial eRNAs involving UCEC. For the key eRNAs in UCEC, we employed RT-qPCR to compare eRNA expression levels in tumor tissues and paired normal adjacent tissues from UCEC patients for validation. Furthermore, the relationships between the key eRNAs and immune activities were measured from several aspects, including the analysis for tumor microenvironment, immune infiltration cells, immune check point genes, tumor mutation burden, and microsatellite instability, as well as m6A related genes. Finally, the key eRNAs were verified by a comprehensive pan-cancer analysis.

**Results:**

IGFBP7 Antisense RNA 1 (IGFBP7-AS1) was identified as the key eRNA for its expression patterns of low levels in tumor tissues and favorable prognostic value in UCEC correlated with its target gene IGFBP7. In RT-qPCR analysis, IGFBP7-AS1 and IGFBP7 had down-regulated expression in tumor tissues, which was consistent with previous analysis. Moreover, IGFBP7-AS1 was found closely related with immune response in relevant immune analyses. Besides, IGFBP7-AS1 and its target gene IGFBP7 correlated with a multi-omics pan-cancer analysis.

**Conclusions:**

Finally, we suggested that IGFBP7-AS1 played a key role in impacting on clinical outcomes of UCEC patients for its possible influence on immune activity.

**Supplementary Information:**

The online version contains supplementary material available at 10.1186/s12575-022-00172-0.

## Introduction

Uterine Corpus Endometrial Carcinoma (UCEC) is the second most prevalent type of malignancy among women worldwide and ranks as the third leading cause of gynecological malignancy death in females. It was estimated there would be 65,620 new cases (following breast cancer, 276,480) and 12,590 deaths (following breast cancer, 42,170; Ovary cancer, 13,940) in 2020 [37]. The death rate for endometrial cancer has increased exceeding 100% during the past 20 years, rising by 8% since 2008 [8]. In China, a total of 9,213 patients died from corpus uteri cancer, accounting for 2.20% of cancer-related deaths from malignancies in females [45]. In recent years, the incidence of and mortality resulted from endometrial cancer has been growing quickly due to the increased cases of obesity and the subsequence hyperinsulinemia [29]. The patients with prognosis for stage I disease who received surgery treatment attended good results, while the treatments for patients with advanced disease (stage III or IV) don’t work well, with overall survival (OS) rates in 5 years ranging from 47 to 69 and 15% to 17%, respectively. Moreover, therapeutic options are still limited except for first-line chemotherapy (Y. C. [23]. Therefore, improvement in UCEC diagnosis is urgently needed.

Long non-coding RNAs (lncRNA) are defined as an important novel class of non-coding RNAs with lengths more than 200 nucleotides that are not translated into protein[34]. lncRNAs are crucial players in regulating chromatin dynamics, gene expresson, growth, differentiation and development [6]. The discoveries of huge numbers of lncRNA, including their wide-range of expression patterns in different types of cancer, their tumor-specificity, and stability in circulating body fluids (plasma and urine) provide a new foundation for developing cancer prognosis and therapies [7]. Also, lncRNAs are also useful in survival outcome prediction of patients. While a lot of lncRNAs have been reported related with UCEC prognosis in many association studies [31, 51], there is little understanding of their function in causing diseases.

Enhancer RNA (eRNA) is a functional subtype of lncRNA transcribed by enhancers, which interacts with target promoter to enhance the transcription of target gene (Z. [50]. eRNAs are widely recognized as bi-direction non-coding RNAs generated by enhancers prevalently bound by RNA polymerase II (Pol II) [17]. There are many eRNAs that show remarkable expression difference between tumor and adjacent normal samples, which raise a potential to target eRNAs for cancer therapies (H. [12],J. H. [22]. The important function of eRNA in oncogene deregulation and cancer initiation has been established in many types of cancer (W. [25]. However, current knowledge about the function and mechanistic roles of eRNA in UECE is limited.

In this study, we aimed to find out the key eRNA and its target gene in UCEC and we identified eRNA IGFBP7-AS1, with its target gene IGFBP7, was significantly correlated with survival outcomes of UCEC patients. Up-regulated levels of IGFBP7-AS1 correlated with increased level of IGFBP7, elevated expression of most immune-related genes and better immune response, which indicated that IGFBP7-AS1 was an immune-related eRNA in favor of clinical outcomes.

## Methods

### Data collection and processing

The RNA expression profile data in pan-cancers (Workflow Type: HTSeq-FPKM) were acquired from The Cancer Genome Atlas (TCGA) database, as well as relevant clinical and survival information [44]. Relevant eRNA information was searched and summarized from relative eRNA references [14]. Then the human gene annotation file was applied to convert the Ensemble transcript IDs of RNA expression profile data into gene symbols. The 1580 eRNAs expression profiles were thus extracted from the RNA expression profile data of UCEC by matching the eRNA gene symbols. The survival-associated eRNAs were identified by Kaplan–Meier tests, taking the *P* < 0.05 as standard cut-off values. The co-expression data was used to calculate the correlations between prognostic eRNAs and corresponding target genes on the basis of Spearman rank correlation coefficients (Spearman’s rank correlation coefficient *R* > 0.5, *p* < 0.05). The most significant ones which closely correlated with both OS survival and predicted target genes were considered as the key eRNAs in UCEC.

Gene Expression Profiling Interactive Analysis (GEPIA) is a well-known online tool for visual analysis from TCGA dataset (http://gepia.cancer-pku.cn/). Besides, it also includes normal and tumor samples of RNA sequencing data from Genotype-Tissue Expression projects [41]. In this study, the comparisons of IGFBP7-AS1 and IGFBP7 expression in normal and tumor samples were visualized by GEPIA.

### Reverse transcription-quantitative (RT-q) PCR for IGFBP7-AS1

Reverse transcription-quantitative (RT-q) PCR was applied to confirm the RNA-sequencing data of IGFBP7-AS1 and IGFBP7. The RNA was extracted from paired tumor and adjacent samples from 12 UCEC patients by TRIzol reagent (Thermo Fisher Scientific). Before reverse transcription to cDNA, 4 × gDNA wiper Mix (Vazyme R323-01), DEPC and total RNA (1 μg) were resuspended and reacted at 42 °C for 2 min to remove the residual genomic DNA from total RNA. Agilent Bioanalyzer 2100 (Agilent Technologies) with RNA 6000 Nano kit was applied to check the integrity of extracted RNA. PrimeScript RT reagent kit was used to synthesize the complementary RNA and SYBR Green PCR Kit (Thermo Fisher Scientific) was employed to conduct real-time quantitative analysis. Glyceraldehyde-3-phosphate dehydrogenase (GAPDH), an enzyme in glycolysis, which is widely distributed in cells of various tissues. GAPDH gene expression changes little in different states, so it was severed as the reference gene, and relative gene expression was estimated by the 2-ΔΔCt (ΔCt = Cttarget gene − Ctinternal control) method. The PCR primer sequences are presented in Table 1.

**Table 1:** Primer sequences of IGFBP7-AS1, IGFBP7 and the reference gene GAPDH

Gene symbol	Forward primer (5′-3′)	Reverse primer (5′-3′)
IGFBP7-AS1	TGGAAAGCTCTTCCTGACCC	TGGTGTGACTTCCGCATGTT
IGFBP7	CGAGCAAGGTCCTTCCATAG	GGTGTCGGGATTCCGATGAC
GAPDH	ATCAATGGAAATCCCATCACCA	GACTCCACGACGTACTCAGCG

### Survival analysis

We performed K-M test to investigate the relationship between the expression of IGFBP7-AS1 and the overall survival time of UCEC patients, as well as pan-cancer patients. Also, K-M curves as the results of survival analysis were generated using “survival” package in R, showing the different survival condition in high and low IGFBP7-AS1 expression groups. Specifically, univariate and multivariate Cox proportional hazard ratio analysis for IGFBP7-AS1 levels and other clinical variables was also applied here with “survival” package, in order to identify the real effects of IGFBP7-AS1 on the prognostic survival of UCEC patients.

### Gene enrichment analysis

To learn about the biological function and related signal pathways of IGFBP7-AS1, GO terms for biology process, cell component, and molecular function and KEGG enrichment pathways were all analyzed by “clusterprofiler” package of R and the results were then visualized into heatmaps through R package “enrichplot” [48].

### Immune microenvironment and immune infiltration analysis

Stromal and immune scores were calculated by ESTIMATE algorithm for each pan-cancer sample and cancer samples were then divided into high and low scores groups by medium stromal and immune scores [47]. In addition, Cell-type identification by estimating relative subsets of RNA transcripts (CIBERSORT), a deconvolution algorithm based on RNA-seq data, was applied to estimate the proportion of 22 immune-infiltration cell types so as to reveal the correlation between IGFBP7-AS1 and immune response [30]. The different infiltration of degrees of immune infiltration lymphocyte types, immune-related pathway and functions in different IGFBP7-AS1 expression groups of UCEC patients were explored by ssGSEA using R package “GSVA”, in order to reflect IGFBP7-AS1’s effect on immune infiltration [4].

### TMB and MSI calculation

Tumor mutation burden (TMB) and microsatellite instability (MSI) information was downloaded from TCGA database. TMB was calculated as somatic mutation incidences per million base pair in the code region of tumor cell genome [9], and MSI was counted by the times of insertion or deletion events that occurred in repeating sequences of genes (Cortes-Ciriano, Lee, Park, Kim, & Park, [15]).

### Drug sensitivity analysis

CellMiner database (https://discover.nci.nih.gov/cellminer/home.do) was used to obtain the drug sensitivity data of different cancer cell lines, which was further filtered by the standard of clinical laboratory verification and FDA certification [36]. Then we used Pearson correlation to figure the connection between IGFBP7-AS1 expression with drug sensitivity.

### Statistical analysis

Statistical analysis was completed with R version 3.6.3 software and related R packages. The gene expression comparisons between different groups were tested by Wilcoxon rank sum test. Spearman rank correlation were used to measure the correlation between IGFBP7-AS1 expression and some targets of research, including target gene expression, TMB, MSI immune cell score and so on. A two-sided *P*-value lower than 0.05 was considered statistically significant. Most of the graphs were plotted through R packages “ggplot2”, and the gene expression comparison plots of paired samples in RT-qPCR were drawn by GraphPad Prism 8.

## Results

### Screening prognosis-associated key eRNAs and target genes in UCEC

**Fig. 1:**
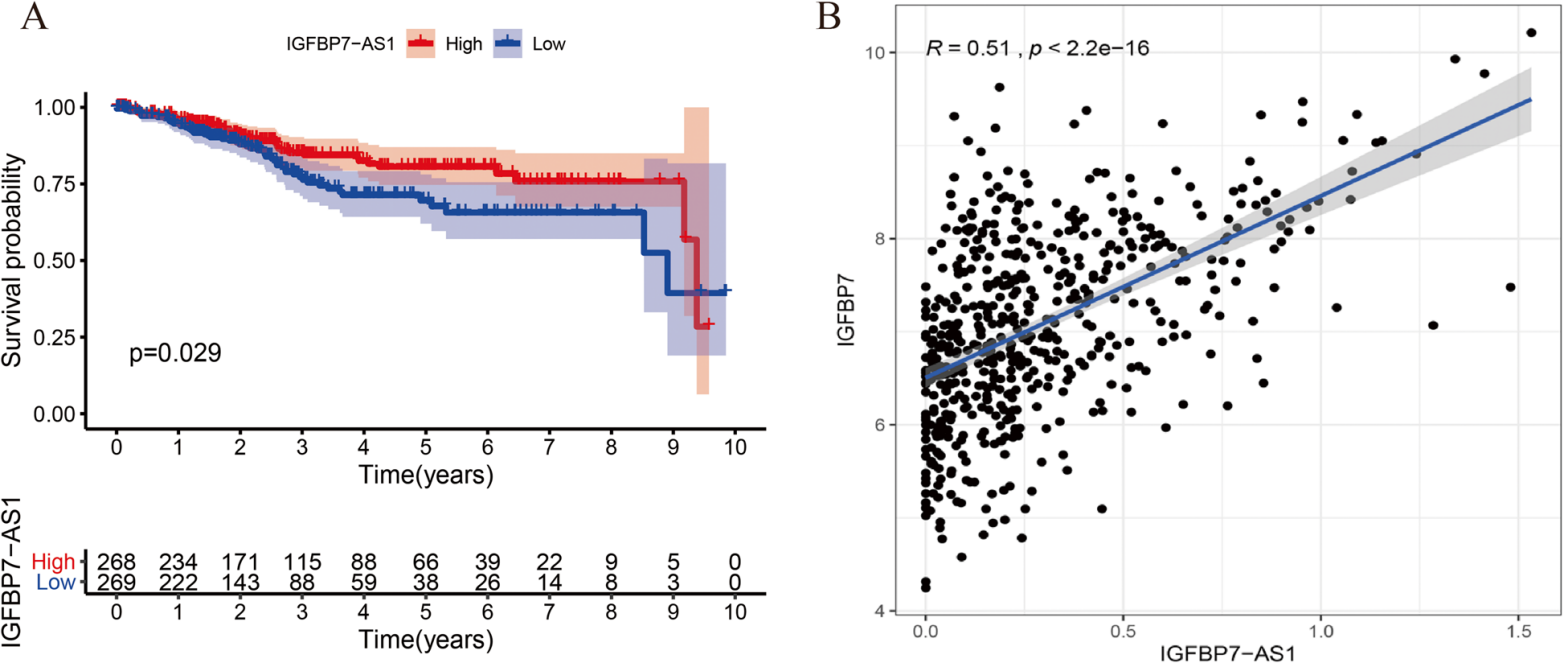
Impact of IGFBP7-AS1 and IGFBP7 on Uterine Corpus Endometrial Carcinoma (UCEC) **A** Kaplan–Meier curve analysis of high-level and low-level IGFBP7-AS1 expression groups **B** Scatter plot showing the significant correlation between IGFBP7-AS1 and IGFBP7 levels

One thousand five hundred eighty eRNA expression data was extracted from the samples of UCEC in TCGA database, as well as the survival and clinical phenotypes dataset, and 120 eRNA gene expressions were found associated with the UCEC patients’ overall survival according to the results from the Kaplan–Meier log-rank test (*p* < 0.05). Spearman rank correlation coefficients were then used here to identify the significant correlations between the prognostic eRNAs and their predicted target genes thus helping with the further screening (Spearman’s rank correlation coefficient *R* > 0.5, *p* < 0.05). Finally, the relevant information of the 48 key eRNAs and their target genes was summarized in Table 2. Especially, the UCEC patients with high expression of eRNA IGFBP7-AS1 showed better overall survival performance compared to those low IGFBP7-AS1 expression ones (Fig. 1A, *p* < 0.05). In addition, as indicated in Fig. 1B, IGFBP7-AS1 had a closely positive correlation with IGFBP7 (*R* = 0.51, *p* < 0.001).

**Table 2:** List of overall survival associated genes derived from enhancers

Symbol	TANRIC Overall Survival Analysis, Log-Rank *p*-Value	Target gene	Correlation between lncRNA and the Neighboring Target	
			**Correlation** **Coefficient R**	***p*-value**
AL031846.1	0.000	APOBEC3H	0.516	< 0.001
LEF1-AS1	0.000	LEF1	0.872	< 0.001
ZFHX4-AS1	0.000	ZFHX4	0.511	< 0.001
SLC16A1-AS1	0.000	SLC16A1	0.655	< 0.001
FZD10-AS1	0.000	FZD10	0.881	< 0.001
CARD18	0.000	CARD18	1.000	< 0.001
SLC47A1P2	0.000	SLC47A1	0.641	< 0.001
KCNK15-AS1	0.000	RIMS4	0.547	< 0.001
TMEM210	0.000	NPDC1	0.539	< 0.001
AL606970.4	0.000	DACT2	0.515	< 0.001
MAL2	0.000	MAL2	1.000	< 0.001
DGCR9	0.001	DGCR9	1.000	< 0.001
AL512306.3	0.001	LRRN2	0.629	< 0.001
AP001189.3	0.003	LRRC32	0.590	< 0.001
FLG-AS1	0.004	FLG	0.732	< 0.001
AL162411.1	0.004	GLDC	0.859	< 0.001
LINC00665	0.004	ZFP82	0.596	< 0.001
MSH6	0.006	MSH6	1.000	< 0.001
LRRC8C-DT	0.007	LRRC8C	0.607	< 0.001
MAP4K3-DT	0.007	MAP4K3	0.544	< 0.001
AC005062.1	0.008	MACC1	0.528	< 0.001
AP000696.1	0.008	SIM2	0.755	< 0.001
LILRP2	0.009	NCR1	0.573	< 0.001
SLC47A1P1	0.010	SLC47A1	0.680	< 0.001
MEG8	0.011	MEG8	1.000	< 0.001
IGFBP7-AS1	0.011	IGFBP7	0.513	< 0.001
CRNDE	0.012	IRX5	0.783	< 0.001
LINC02418	0.012	FZD10	0.629	< 0.001
LINC01833	0.015	SIX3	0.723	< 0.001
LINC00261	0.017	FOXA2	0.909	< 0.001
CCDC144NL-AS1	0.019	CCDC144NL	0.551	< 0.001
GAS1RR	0.020	GAS1	0.740	< 0.001
GRAMD1B	0.020	GRAMD1B	1.000	< 0.001
AL357153.1	0.021	TTC9	0.601	< 0.001
GACAT3	0.022	MYCN	0.612	< 0.001
CDKN2B-AS1	0.022	CDKN2A	0.728	< 0.001
AC007255.1	0.025	PRR15	0.745	< 0.001
AP001042.1	0.025	ETS2	0.607	< 0.001
AC022424.1	0.028	ADAMTS16	0.699	< 0.001
AC025871.2	0.028	FBXO16	0.500	< 0.001
AC092894.1	0.030	CD200	0.676	< 0.001
LINC01411	0.037	MSX2	0.728	< 0.001
LINC02381	0.038	HOXC4	0.707	< 0.001
LINC02389	0.041	TBC1D30	0.610	< 0.001
WT1-AS	0.041	WT1	0.915	< 0.001
SLC44A3-AS1	0.042	SLC44A3	0.717	< 0.001
AL353747.3	0.046	UNC93A	0.587	< 0.001
GATM	0.049	GATM	1.000	< 0.001

### Data validation of IGFBP7-AS1 and IGFBP7 levels by reverse transcription quantitative PCR (RT-qPCR)

**Fig. 2:**
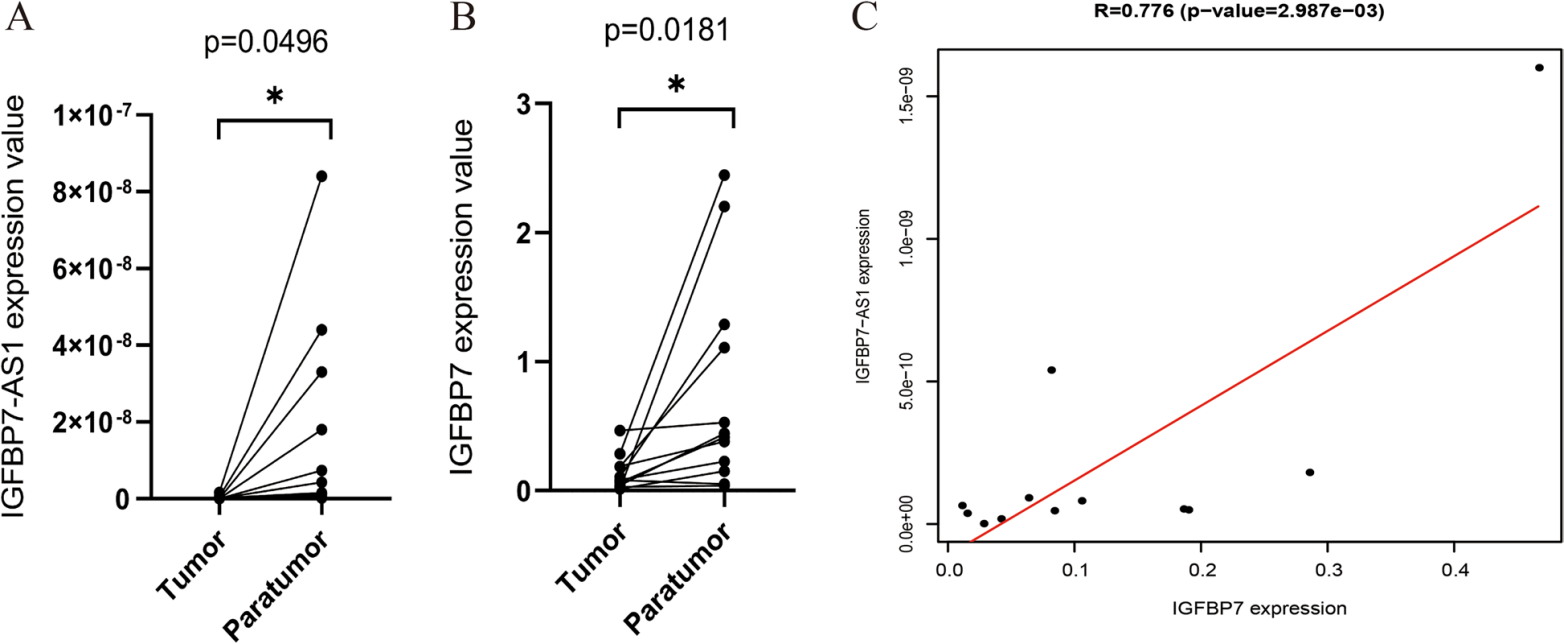
Quantification of IGFBP7-AS1 and IGFBP7 levels by RT-qPCR. **A**, **B** Expression levels of IGFBP7-AS1 and IGFBP7 in UCEC samples and paired adjacent samples. **C** Significant correlation between IGFBP7-AS1 and IGFBP7 levels in tumor samples

qRT-PCR was used to measure the expression levels of IGFBP7-AS1 and IGFBP7 in UCEC samples and paired adjacent samples. Down-regulation of IGFBP7-AS1 and IGFBP7 was seen in tumors when comparing it with adjacent samples, in accordance with its expression trend in the TCGA dataset (Fig. 2A, B). A significantly positive correlation was seen between these two genes in tumor tissues (*R* = 0.776, *p* < 0.001, Fig. 2C).

### Correlation of IGFBP7-AS1 expression with clinical features and prognosis survival of UCEC

**Fig. 3:**
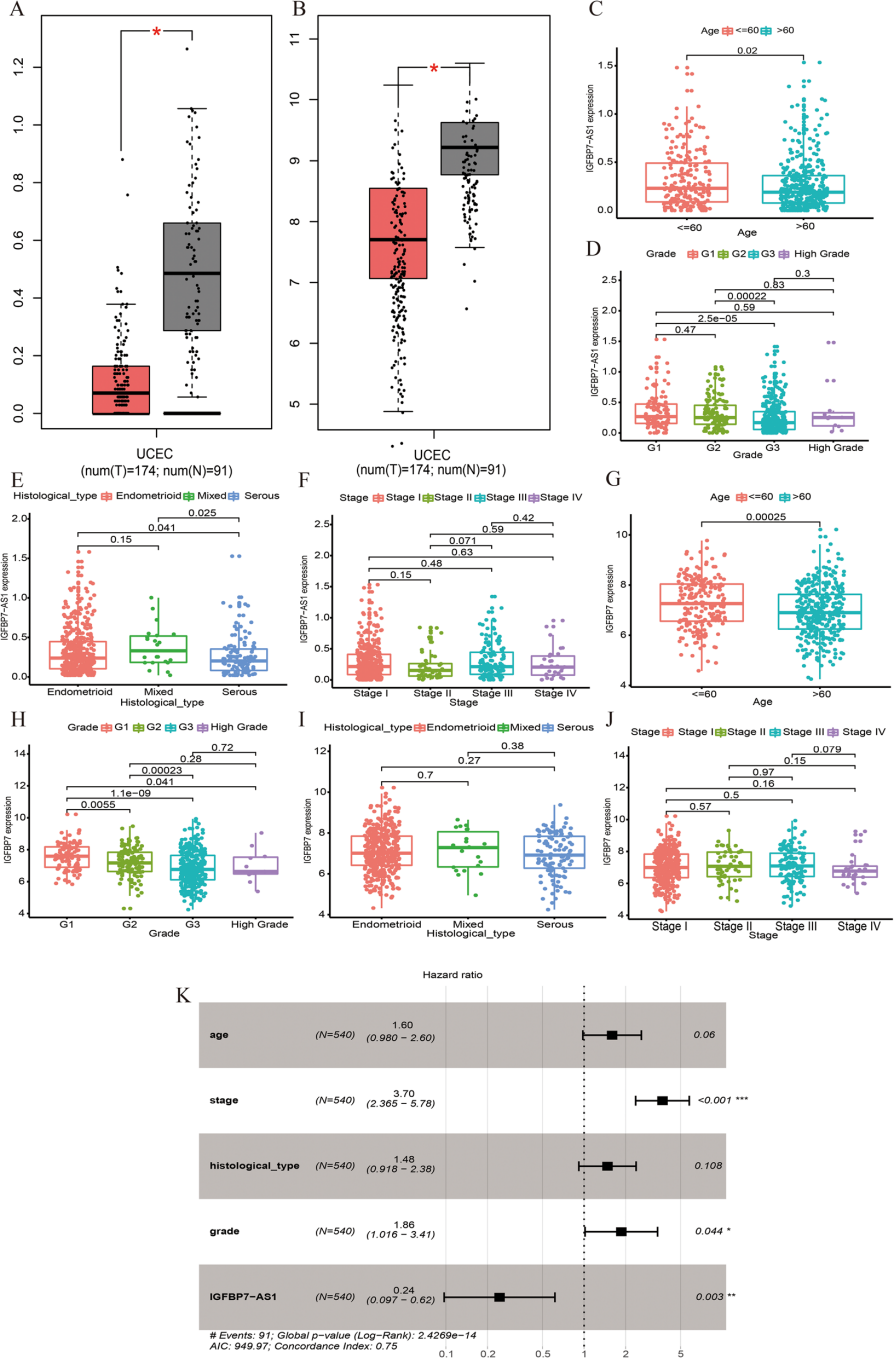
IGFBP7-AS1 and IGFBP7 expression in UCEC and their correlations with various clinicopathology features and prognosis survival. **A** IGFBP7-AS1 expression in UCEC tumor and normal tissues (the red for tumor tissues and the black for normal tissues). **B** IGFBP7 expression in UCEC tumor and normal tissues (the red for tumor tissues and the black for normal tissues). **C** The expression of IGFBP7-AS1 in different age, **D** tumor grade, **E** histological type and **F** clinical stage. **G** The expression of IGFBP7 in different age, **H** tumor grade, **I** histological type and **J** clinical stage. **K** forest plot of Multivariate COX regression analysis about age, stage, histological type, tumor grade and IGFBP7-AS1 expression

Using data from GEPIA database, we compared the IGFBP7-AS1 and IGFBP7 expression levels across UCEC normal and cancer tissues, and it was clear that they all had decreased expression in tumor tissue (Fig. 3A, B). Subsequently, we explored the relationship of IGFBP7-AS1 and IGFBP7 expression with clinicopathologic variables of UCEC and found that the expression level of IGFBP7-AS1 was significantly different in different age (Fig. 3C), tumor grade (Fig. 3D) and histological type (Fig. 3E) group; however, clinical stage showed no significant correlation with IGFBP7-AS1 levels (Fig. 3F). Besides, IGFBP7 was found related to patient age (Fig. 3G), tumor grade (Fig. 3H), but had no evidence for its correlation with histological type (Fig. 3I) and clinical stage (Fig. 3J). Using Cox regression, we further investigated the relationship between IGFBP7-AS1 and OS, along with the clinicopathologic characters before. The results were listed in Table 3, and some parameters, including clinical stage, tumor grade and IGFBP7-AS1 levels, turned out to be linked with OS both in univariate and multivariate regression. Figure 3K, showing a forest boxplot from Multivariate analysis, suggested that mRNA IGFBP7-AS1 was an independent favorable prognostic factor to UCEC. Since the variable age and histological type were insignificant in the multivariate analysis, another multivariate Cox model with only the significant ones of clinical stage, tumor grade and IGFBP7-AS1 levels was conducted to verify the results, in which all the three variables remained to be significant, supporting the finding of IGFBP7-AS1’s independent prognosis for UCEC (Supplementary Table 1).

**Table 3:** Univariate analysis and multivariate analysis for the association of IGFBP7-AS1 expression with OS of UCEC patients

Parameter	Univariate analysis				Multivariate analysis			
	**HR**	**HR.95L**	**HR.95H**	***p*value**	**HR**	**HR.95L**	**HR.95H**	***p*value**
age	1.807	1.133	2.884	0.013	1.596	0.980	2.601	0.060
stage	3.925	2.590	5.949	1.16E-10	3.697	2.365	5.780	9.75E-09
histological type	2.880	1.903	4.358	5.64E-07	1.479	0.918	2.382	0.108
grade	3.436	2.002	5.899	7.58E-06	1.861	1.016	3.409	0.044
IGFBP7-AS1	0.285	0.109	0.743	0.010	0.245	0.097	0.616	0.003

### Enrichment analysis

**Fig. 4:**
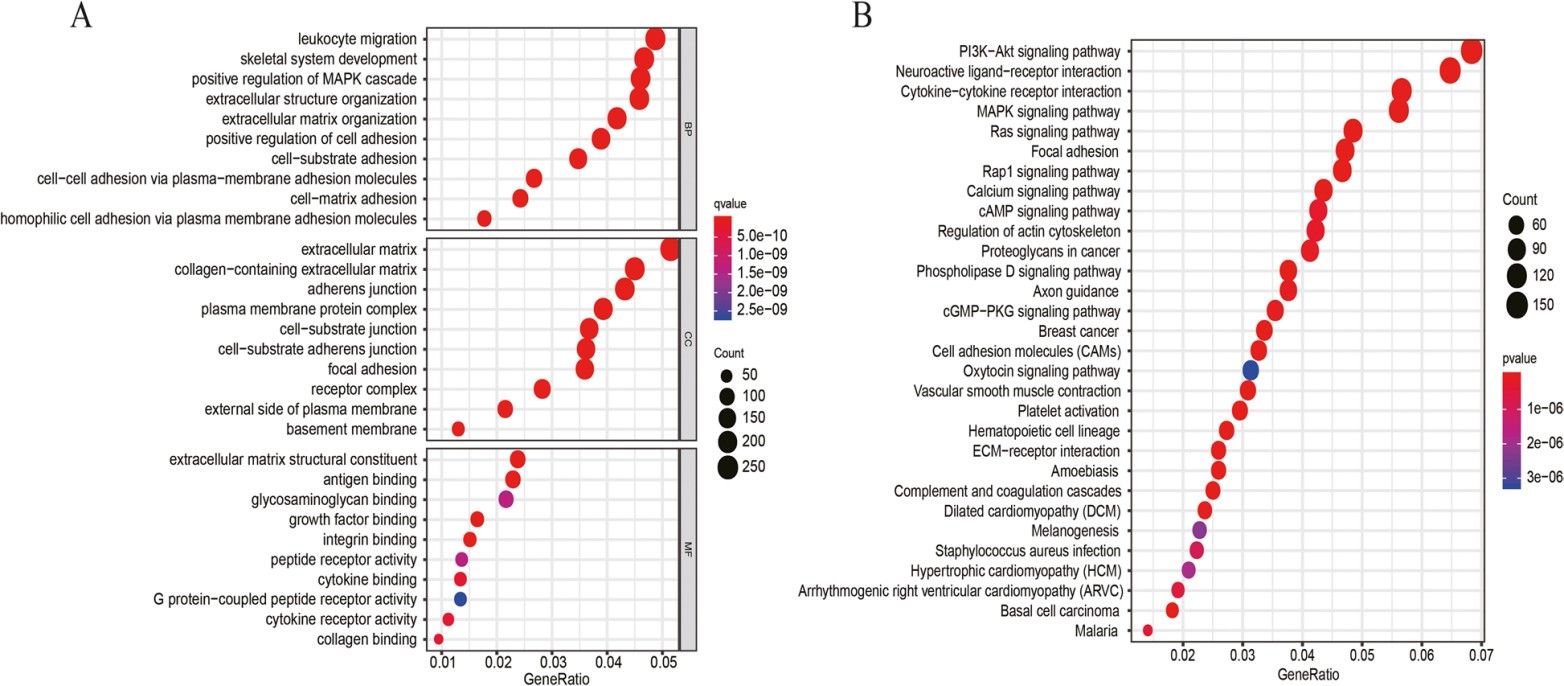
GO term and KEGG pathway analysis in UCEC. **A** bubble plot of GO term analysis **B** of KEGG pathway analysis

GO enrichment analysis and KEGG pathway analysis were performed here so as to provide more information about the biological function of IGFBP7-AS1. Figure 4A, B were the bubble plots about the GO terms and KEGG signaling pathways of IGFBP7-AS1. Top 10 terms for biological process (BP), cellular component (CC), molecular function (MF) was extracted as the most important ones associated with IGFBP7-AS1——leukocyte migration in BP and extracellular matrix in CC were demonstrated to have strong correlation with IGFBP7-AS1. The highest-ranking signaling pathways in KEGG were PI3K − Akt signaling pathway, Neuroactive ligand − receptor interaction, Cytokine − cytokine receptor interaction and MAPK signaling pathway, which means IGFBP7-AS1 may regulate leukocyte migration through these pathways to influence on the occurrence and development of tumors. Moreover, Neuroactive ligand − receptor interaction and Cytokine − cytokine receptor interaction were all immune related pathways, which suggested the connection between IGFBP7-AS1 and immune activities.

### IGFBP7-AS1 expression correlated with immune status

**Fig. 5:**
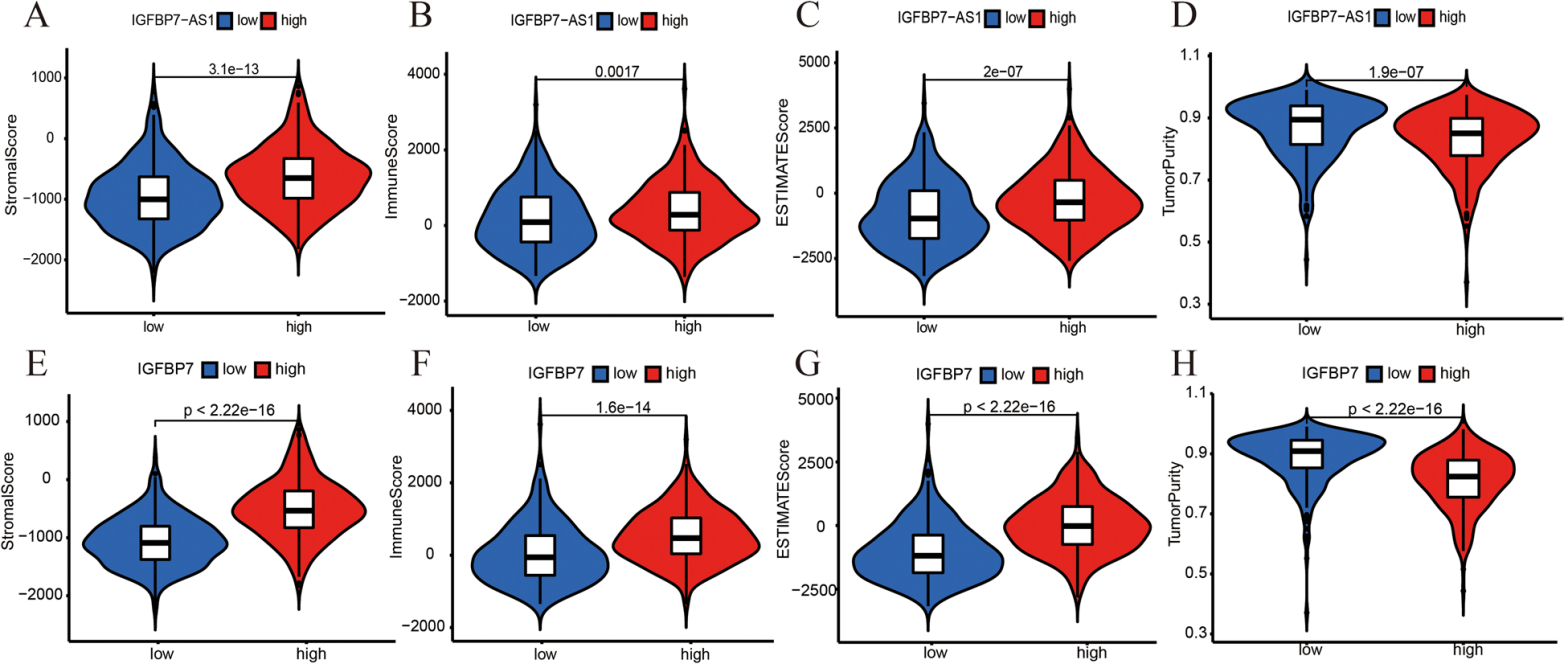
TME analysis for IGFBP7-AS1 and IGFBP7. **A** The IGFBP7-AS1 expression differences in Stromal score, **B** Immune score, **C** ESTIMATE score and **D** Tumor purity. **E** The IGFBP7 expression differences in Stromal score, **F** Immune score, **G** ESTIMATE score and **H** Tumor purity

The tumor immune microenvironment (TME) plays an essential role in activating heterogeneity among cancer cells, thus stimulating multidrug resistance and resulting in the occurrence, development, and metastasis of tumors. The tumor immune microenvironment has been a critical part in tumor research so we calculated the stromal score, immune score, ESTIMATE score and tumor purity of each tumor sample by ESTIMATE and compared them in high and low IGFBP7-AS1 and IGFBP7 expression groups. A high stromal and immune score or low tumor purity usually contributed to better prognosis and response to immunotherapy. The results indicated that increased IGFBP7-AS1 and IGFBP7 expression was closely related with higher stromal score, immune score, estimate score and lower tumor purity (Fig. 5A-H). So high levels of IGFBP7-AS1 and IGFBP7 may be beneficial for immunotherapy effect of UCEC.

**Fig. 6:**
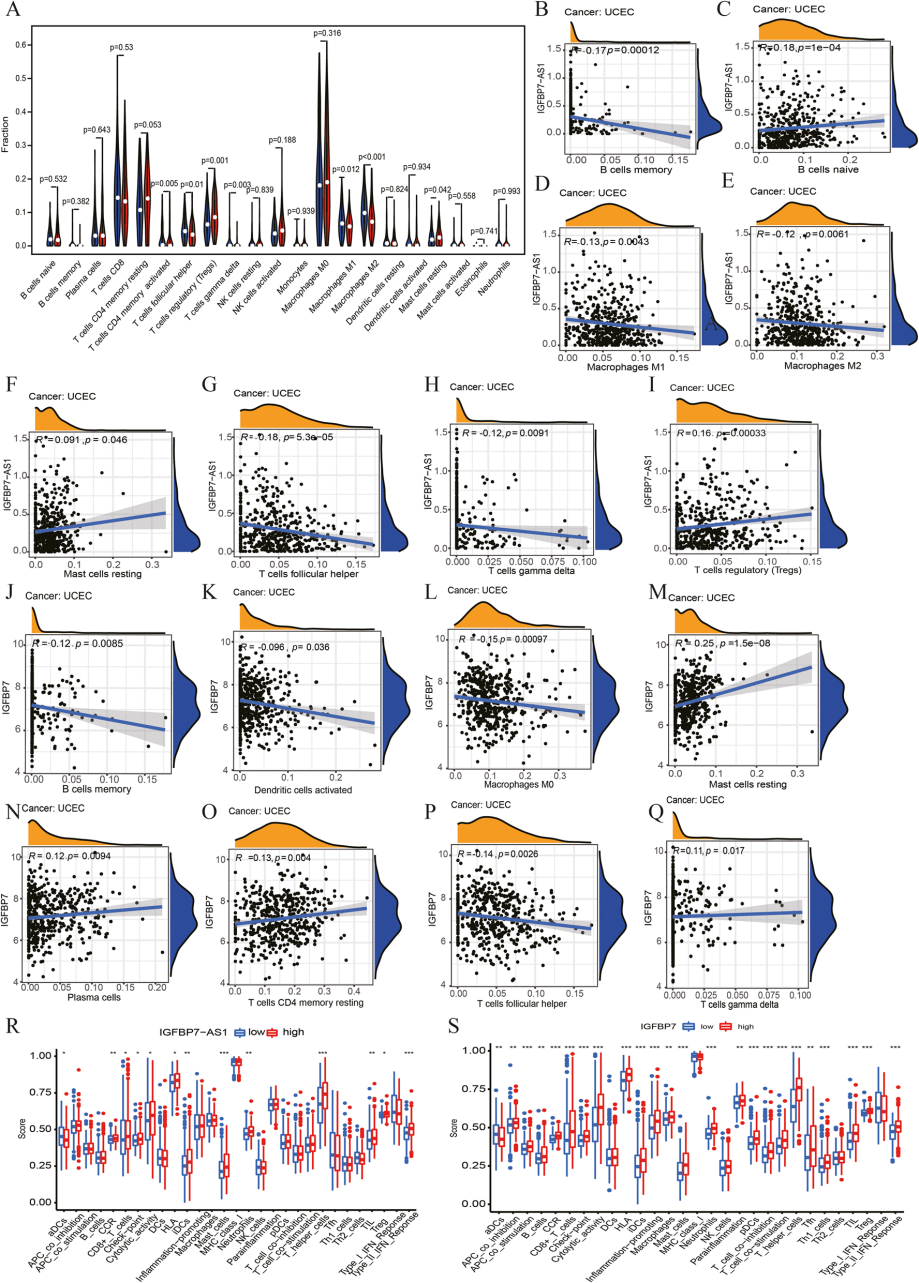
Relationship of IGFBP7-AS1 expression with immune related cells and immune infiltration scores of TILs and immune-related substances. **A** The boxplots of immune cells proportion in different IGFBP7-AS1 expression groups (the red for high expression group and the blue for low expression group of IGFBP7-AS1). **B** Scatterplot of IGFBP7-AS1 correlated with B Cells memory, **C** B cells native, **D** Macrophages M1, **E** Macrophages M2, **F** Mast cells resting, **G** T cells follicular helper, **H** T cells gamma delta and **I** T cells regulatory (Tregs). **J** Scatterplot of IGFBP7 correlated with memory B cells, **K** Dendritic cells activated, **L** Macrophages M0, **M** Mast cells resting, **N** Plasma cells, **O** T cells CD4 memory resting, **P** T cells follicular helper and **Q** T cells gamma delta. **R** The boxplots of immune infiltration cell score in different IGFBP7-AS1 and **S** IGFBP7 expression groups

To have a better knowledge of their association with immune related cells, we employed CIBERSORT to further process the biological role of IGFBP7-AS1 in TME. Results in boxplot of Fig. 6A indicated that T cells CD4 memory activated, T cells follicular helper, T cells regulatory (Tregs), T cells gamma delta, Macrophages M1, Macrophages M2 and Mast cell resting were the immune cells affected by IGFBP7-AS1 expression. Among them, T cells CD4 memory activated, T cells follicular helper, T cells gamma delta, Macrophages M1 and Macrophages M2 showed low proportion in the high IGFBP7-AS1 expression groups compared to the low. In contrast, T cells regulatory (Tregs) proportion and Mast cell resting were clearly up-regulated in high-level IGFBP7-AS1 group.

We further investigated the detailed correlation between immune cells and expression of IGFBP7-AS1 and IGFBP7. Levels of IGFBP7-AS1 expression were inversely correlated with memory B cells, Macrophages M1, Macrophages M2, T cells follicular helper and T cells gamma delta, whereas native B cells, Mast cells resting and T cells regulatory (Tregs) were positively associated with IGFBP7-AS1 expression (Fig. 6B-I). At the same time, IGFBP7 expression levels were positively correlated with levels of Mast cells resting, Plasma cells, T cells CD4 memory resting and T cells gamma delta, and diversely correlated with memory B cells, Dendritic cells activated, Macrophages M0 and T cells follicular helper (Fig. 6J-Q).

Tumor-infiltrating lymphocytes (TILs) play a key role in sentinel lymph node status and overall survival rate prediction [19]. Immune infiltration scores of TIL types and immune-related substances were calculated in order to determine if the IGFBP7-AS1 and IGFBP7 connected with the immune infiltration. We assessed differences between high- and low- levels in immune infiltration cell and marked differences were shown in aDCs, CCR, CD8 + T cells, Check point, Cytolytic activity, HLA, iDCs, Mast cells, Neutrophils, T helper cells, TIL, Treg and Type II IFN Reponses between IGFBP7-AS1 subgroups. Except DCs, MHC class I, NK cells, Th2 cells and Type I IFN Response, IGFBP7 expressed significantly different in other immune cells (Fig. 6R, S).

### IGFBP7-AS1 expression correlated with immune checkpoint genes expression

**Fig. 7:**
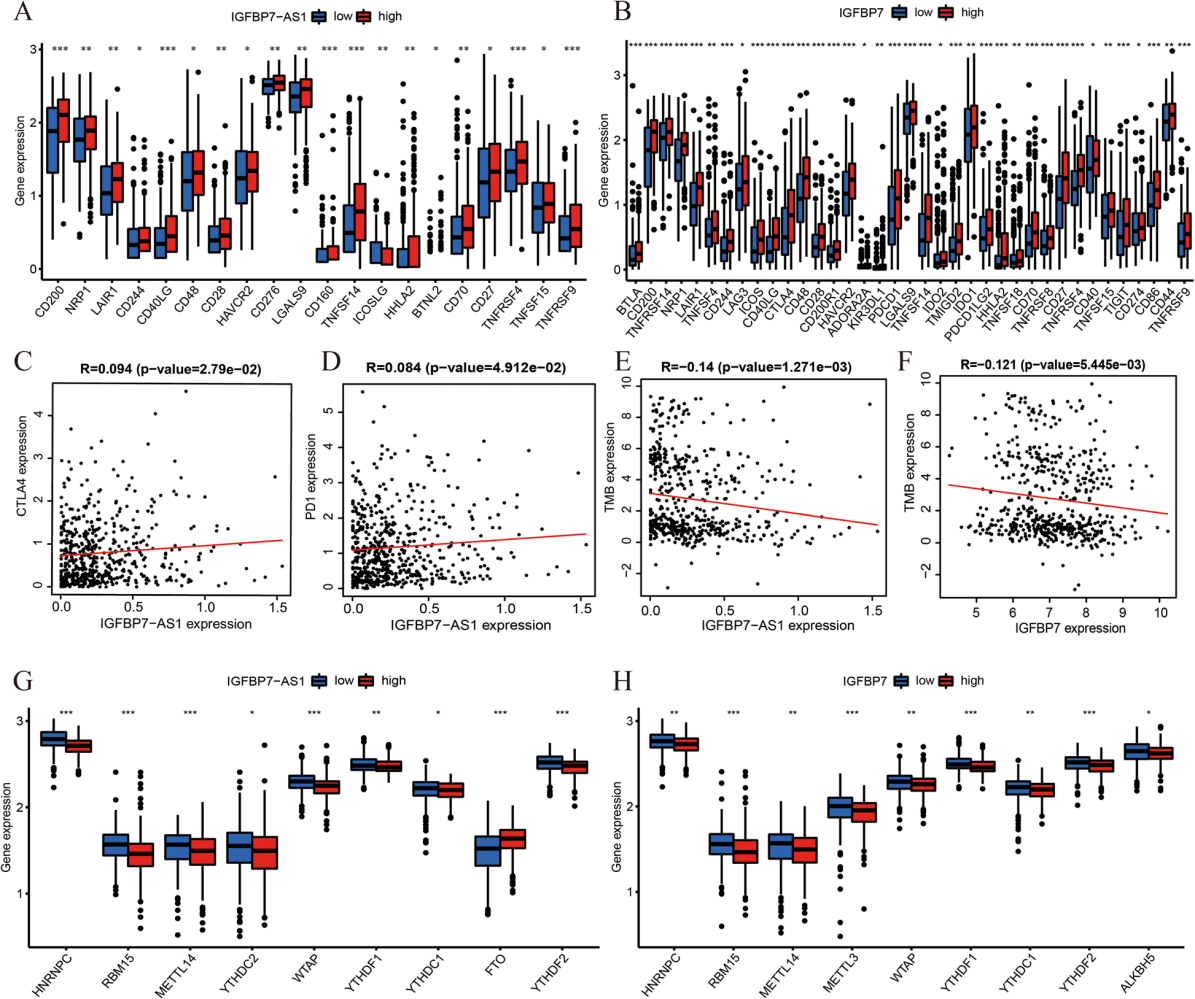
Correlations of IGFBP7-AS1 and IGFBP7 with immune checkpoint genes, TMB and m6A genes. **A** The immune checkpoints related genes expression in different IGFBP7-AS1 and **B** IGFBP7 expression groups. **C** The scatter plot showing the correlation between IGFBP7-AS1 and CTLA4 levels. **D** The scatter plot showing the correlation between IGFBP7-AS1 and PD1 levels. **E** The correlation scatterplot of TMB with IGFBP7-AS1 expression and **F** IGFBP7 expression. **G** The m6A genes expression in different IGFBP7-AS1 and **H** IGFBP7 expression groups

Then, we analyzed the immune checkpoint genes expression with IGFBP7-AS1 and IGFBP7. We could find in the pictures plotted in Fig. 7A, B, that these immune checkpoints related genes (CD200, NRP1, LAIR1, CD244, CD40LG, CD48, CD28, HAVCR2, TNFSF14, HHLA2, CD70, CD27, TNFRSF4, TNFSF15, TNFRSF9) expressed differently between high- and low-expression groups of both IGFBP7-AS1 and IGFBP7. PD-1 is a protein on the surface of T and B cells that acts as an essential role in regulating the immune system's response to the cells in the human body by down-regulating the immune system and developing self-tolerance by suppression of T cell inflammatory activity [20]. CTLA4 is a protein receptor that works as a member of immune checkpoints and has effect on immune responses downregulation [32]. They were essential immune checkpoints in carcinoma proliferation research and immune checkpoint blockade therapy by targeting PD-1and CTLA4 revealed promising clinical effects. These key modulators were detected to express more accompanied with higher IGFBP7-AS1 levels (Fig. 7C, D).

### IGFBP7-AS1 Had a Significant Negative Correlation with TMB

TMB (tumor mutational burden) is a genetic characteristic of tumorous tissue that can provide valuable information for our cancer study and relevant treatment. It is defined as the number of non-inherited mutations per Mb of investigated genomic sequence and plays a key role in cancer prognosis and response to tumor immunotherapy treatment [11]. We calculated the TMB and discovered its connection with IGFBP7-AS1 and IGFBP7. The results suggested that both of IGFBP7-AS1 and IGFBP7 had a significant negative correlation with TMB. The more expression of IGFBP7-AS1 or IGFBP7, the less expression of TMB (Fig. 7E, F).

### IGFBP7-AS1 expression correlated with m6A genes expression

m6A(6-methyladenine), a common type of mRNA methylation, impacts on the cancer development for its versatile functions in various physiological processes [38]. The connections between m6A and numerous cancer types have been indicated in many reports involving breast cancer, prostate cancer, stomach cancer and so on. There are some genes (HNRNPC, RBM15, METTL14, YTHDC2, WTAP, YTHDF1, YTHDC1, FTO, YTHDF2) proved to regulate the modification levels of m6A and then affect the cancer cell proliferation. These genes all expressed variously in different IGFBP7-AS1 and IGFBP7 levels groups (Fig. 7G, H). Except for FTO with IGFBP7-AS1, most of the m6A genes were in down-expressed condition with high levels of IGFBP7-AS1 and IGFBP7.

### Pan-cancer verification of IGFBP7-AS1 and IGFBP7 levels

Comprehensive omics analysis of IGFBP7-AS1 across 33 cancers was conducted to verify IGFBP7-AS1’s role in diverse cancers and except to provide robust evidence for potential tumor research. We obtained the expression of IGFBP7-AS1 and IGFBP7 across 33 cancer samples and respective normal tissues from TCGA project. IGFBP7-AS1 and IGFBP7 expression differences could be found in BLCA, BRCA, CESC, CHOL, COAD, GBM, HNSC, KICH, KIRC, KIRP, LUAD, LUSC, PRAD, THCA and UCEC. Besides, IGFBP7 levels differed in SARC, STAD and IGFBP7-AS1 differed in LIHC as well (Supplementary Fig. 1A, B).

Next, we identified the prognostic value of IGFBP7 − AS1 for pan-cancer. Patients in the high-expression group survived longer than those in the low-expression group in LAML, LUAD and UCEC and the outcomes of LGG, MESO and STAD were opposite in Kaplan–Meier survival curves (Supplementary Fig. 1C-H). Furthermore, univariate Cox proportional hazard regressions were modeled to understand the altered expression of IGFBP7 − AS1 and IGFBP7 with patient overall survival, and the directions of prognostic effect were manifested varied depending on cancer types (Supplementary Fig. 1I).

**Fig. 8:**
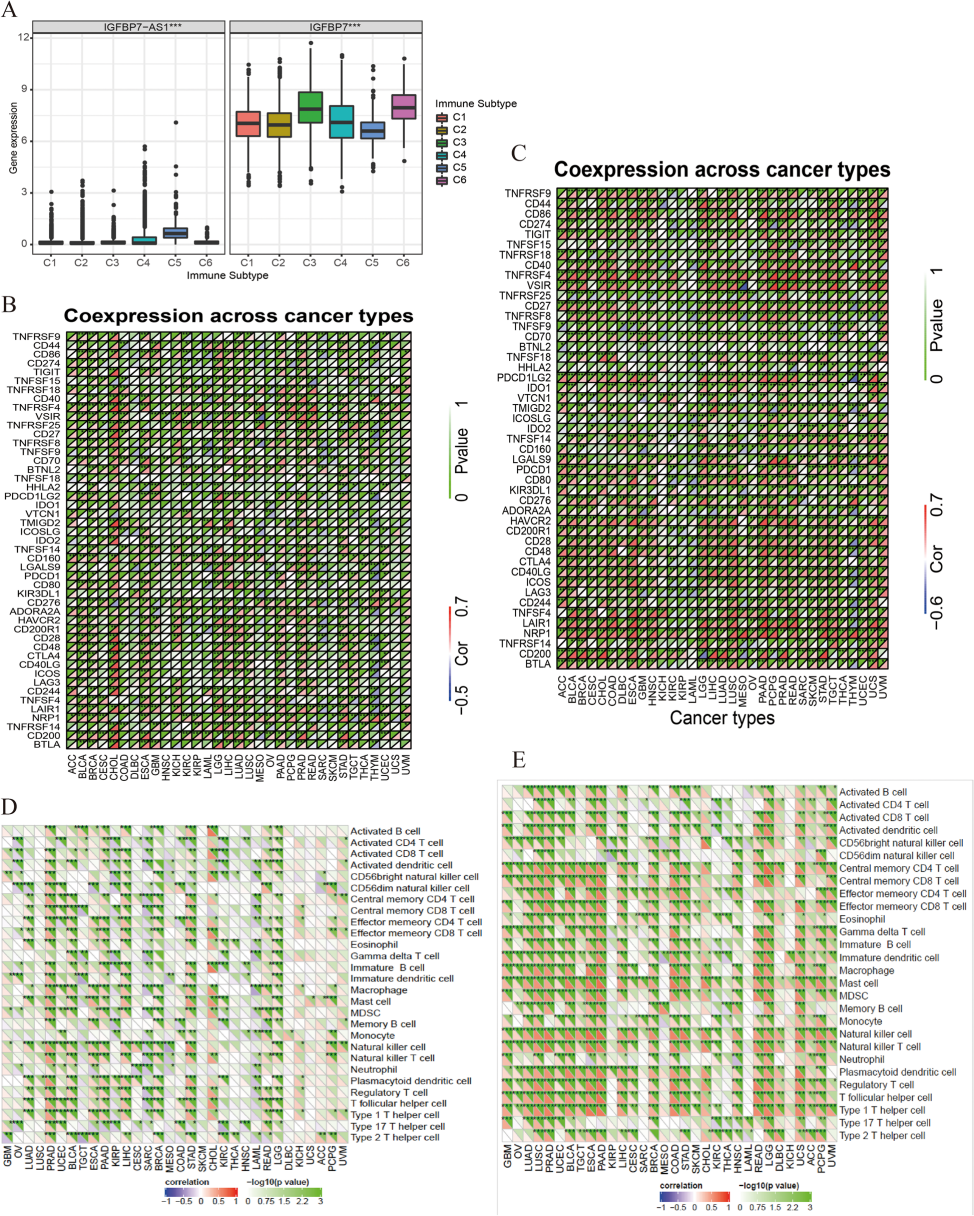
Correlations with immune subtypes, immune checkpoints and TILs. **A** Correlation between gene expression and immune infiltration subtypes in patients of pan-cancer. **B**, **C** Heatmaps about the correlation between IGFBP7-AS1 and IGFBP7 expression levels and acknowledged immune checkpoints’ mRNA expression. **D**, **E** Heatmaps about the relationship between IGFBP7-AS1 and IGFBP7 expression and immune infiltration cells

We first investigated the association of IGFBP7 − AS1 and IGFBP7 expression with six subtypes of immune infiltration: C1 (wound healing), C2 (INF-r dominant), C3 (inflammation), C4 (lymphopenia dominant), C5 (immunologically quiet), and C6 (TGFβ dominant) [40]. Both IGFBP7 − AS1 and IGFBP7 were strongly correlated with immune subtypes in pan-cancer (Fig. 8A). Next, we evaluated whether a link between IGFBP7 − AS1 and IGFBP7 and expression of genes recognized as checkpoint components existed. Co-expression with immune checkpoint related genes in pan-cancers was exhibited in heatmaps of Fig. 8B, C. High positive correlations could be seen in tumors like CHOL, ESCA, LGG, LIHC, LUAD, LUSC and so on while negative ones were mainly appeared in MESO, LAML and THYM.

Also, the relationship with immune infiltration cells were analyzed here, and results were presented in Fig. 8D, E, which suggested that IGFBP7 − AS1 and IGFBP7 were strongly associated with immune cells and most of the correlations were positive.

**Fig. 9:**
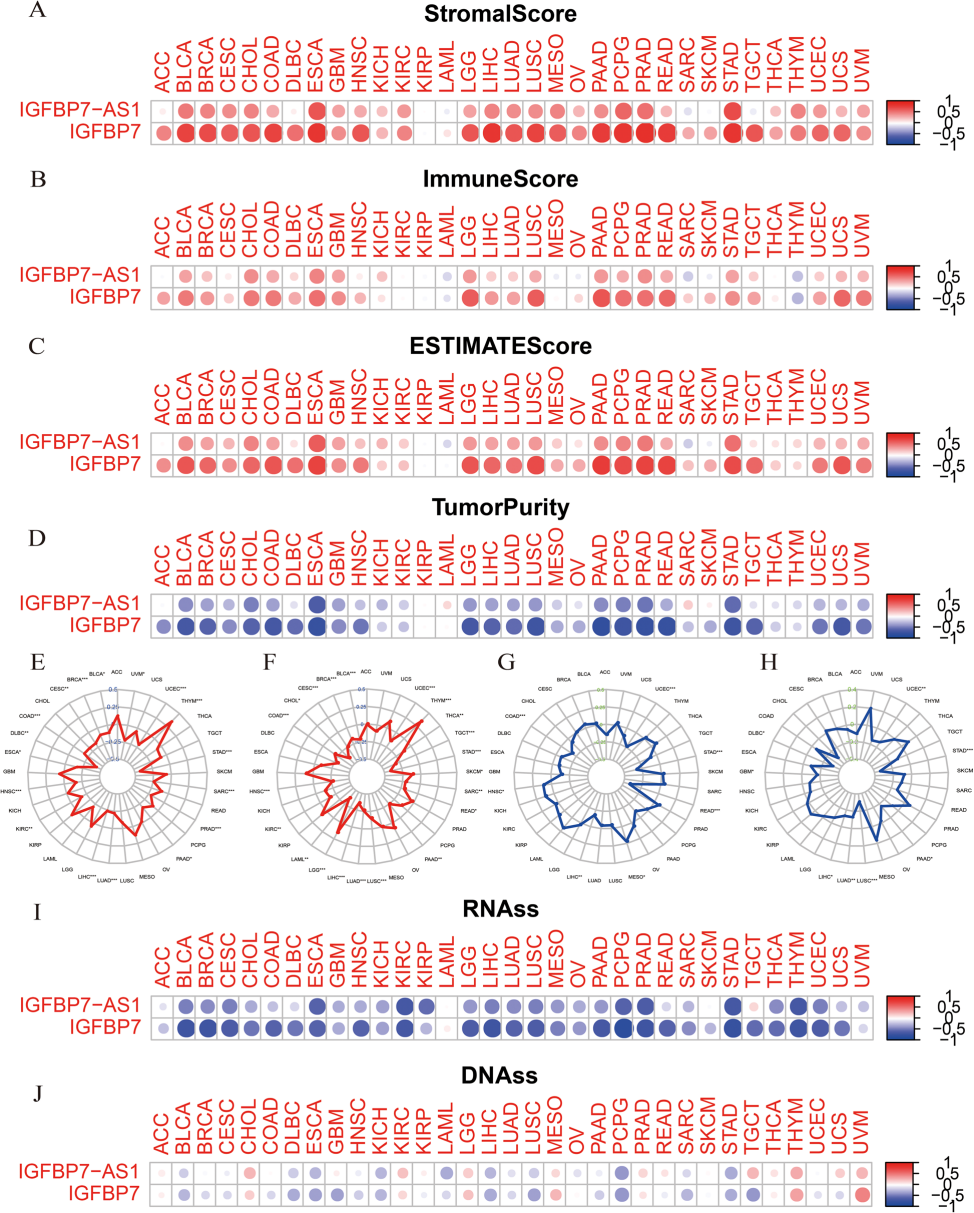
Correlations of IGFBP7-AS1 and IGFBP7 expression with TME, TMB, MSI and tumor stem cells in multiple cancer. **A** The relationship between gene expression and stromal score, **B** immune score, **C** ESTIMATE score and **D** tumor purity. **E**, **F** Correlation between TMB and IGFBP7-AS1 and IGFBP7 expression. **G**, **H** Correlation between MSI and IGFBP7-AS1 and IGFBP7 expression. **I** The relationship between gene expression and RNAss and (J) DNAss

We have done TME analysis of IGFBP7 − AS1 and IGFBP7 in UCEC, so in this section we intended to confirm IGFBP7 − AS1 and IGFBP7’s role in tumor progression and immune response in pan-cancer. Stromal, immune, ESTIMATE score and tumor purity of 33 pan-cancer were shown in Fig. 9A-D. IGFBP7 − AS1 and IGFBP7 expression were positively relevant to stromal score in majority pan-cancers with exceptions of SARC and STAD. In immune score analysis, IGFBP7 − AS1 and IGFBP7 still showed positive correlation with immune score across most of cancers, with the obvious exception of THYM, LAML, and IGFBP7 − AS1 in MESO, OV, SARC, SKCM. Up-level of ESTIMATE score and down-level tumor purity usually came with high levels of IGFBP7 − AS1 and IGFBP7(exceptions were TGCT, LAML, SARC in ESTIMATE score or tumor purity).

After that, we focused on the association with TMB and MSI across 33 pan-cancer and the correlation was exhibited in radar plot Fig. 9E-H. Most of the cancer including BLCA, BRCA, CESC, COAD, DLBC, HNSC, KIRC, LIHC, LUAD, PAAD, PRAD, SARC, STAD, UCEC, UVM had a significantly negative correlation with IGFBP7 − AS1 in terms of TMB, apart from THYM, which showed reverse result with significance. As for IGFBP7, its expression was significant associated with TMB in 21 out of 33 cancers, and most of the trends were similar with IGFBP7 − AS1. The results were consistent with the TMB analysis in UCEC.

MSI (microsatellite instability) is a good marker for determining a prognosis for cancer treatments, and the elevated MSI may be an indicator of higher tumor risk for the reason that the tumor’s disrupted function increases the gene instability [16]. The MSI radar plots reflected that in COAD, LIHC, STAD, as well as UCEC, IGFBP7 − AS1 was acted as negative correlation with MSI, contrary to the condition in HNSC and MESO. While for IGFBP7, the significant associations with MSI turned out to be all negative in DLBC, GBM, LIHC, LUAD, LUAC, PAAD, STAD and UCEC.

Expression-based RNA stemness score (RNAss) and methylation-based DNA stemness score (DNAss) were used to reflect the features of tumor stem cells [28]. Higher stemness index values are associated with stimulate biological processes in tumor stem cells and promoted tumor dedifferentiation [5]. At the same time, we found IGFBP7 − AS1 and IGFBP7 were negatively correlated with RNAss and DNAss across most of the cancers (Fig. 9I, J), and a decrease in tumor stemness was usually linked with better survival. All these evidences can help to explain the IGFBP7 − AS1 and IGFBP7’s mechanisms in modulating the overall survival.

Using CellMiner database, we assessed the influence of IGFBP7 − AS1 and IGFBP7 on drug sensitivity, and such research could help us to provide better precision treatment for patients. We chose the significantly relevant ones according to *P* values here. With the increase in the expression of IGFBP7 − AS1, the less sensitive the cells were to chemotherapeutic drugs involving Veliparib, Mocetinostat, CYC-116 and Alisertib (Supplementary Fig. 2).

## Discussion

Insulin-like growth factor-binding protein 7(IGFBP7) is a new member of the IGFBPs gene family and IGFBP7 Antisense RNA 1(IGFBP7-AS1) is a related RNA gene affiliated with lncRNA class. There were already several researches providing resources for exploring the mechanism of IGFBP7-AS1 in tumorigenesis. One of them revealed IGFBP7-AS1 might have influence on glioma cell survival by regulating tumor growth and migration. In addition, further functional supported experiments demonstrated the knockdown of IGFBP7-AS1 inhibited the invasion and viability of U87 and U251 glioma cells (D. [24]. There were also results indicating that Epstein Barr-virus (EBV) may dysregulate host lncRNAs like IGFBP7-AS1 to regulate its own replication, thus facilitating tumorigenesis [27]. Although the IGFBP7-AS1’s intervention in the progression of tumor proliferation and migration of few specific cancer types has been confirmed, we still lack of the mechanistic explanations for the prognostic ability of IGFBP7-AS1 in majority cancers, not to mention in UCEC. Also, in-depth researches from a clinical perspective are still waiting to be conducted. It may therefore become a novel target for future molecular therapy development.

In our study, IGFBP7-AS1 was determined as the key eRNA of UCEC with its target gene IGFBP7 according to the criteria we set. High expression group of IGFBP7-AS1 was related to better clinical traits, and IGFBP7-AS1 had decreased expression in tumor samples. IGFBP7-AS1 was also identified as an independent protective factor for the clinical outcome of UCEC patients by both univariate and multivariate cox regression, in accordance with the results from Kaplan–Meier survival curves. All this evidence supported that reduced IGFBP7-AS1 expression was associated with unfavorable prognosis in UCEC patients. UCEC patients with low IGFBP7-AS1 expression were more likely to present a more advanced tumor grade and age group, which was the same with IGFBP7. Thus, we suggested that IGFBP7-AS1 had a potential influence in UCEC progression.

Insulin Growth Factor Binding Protein 7 (IGFBP7) is a protein belonging to IGFBP superfamily. There is evidence indicating that IGFBPs function as transporters of the insulin-like growth factors (IGF), lengthen their half-time and regulate their access to their receptors, thereby affecting cell growth, differentiation and metabolic processes in human body [21]. IGFBP7 is a crucial player responsible for tumorigenesis and tumor progression. Accumulating studies clarified that IGFBP7 regulated varied in diverse types of malignancies. Its importance in many tumors like breast cancer, gastric cancer and lung tumors has been revealed in a lot of previous studies (Y. [13, 21, 39].

GO terms analysis revealed upregulated IGFBP7-AS1 was to be primarily connected with leukocyte migration, skeletal system development and extracellular matrix. Meanwhile, KEGG pathways analysis showed five pathways including PI3K − Akt signaling pathway, Neuroactive ligand − receptor interaction, Cytokine − cytokine receptor interaction, MAPK signaling pathway and Ras signaling pathways were showed significantly correlated with IGFBP7-AS1. PI3K − Akt signaling pathway has been demonstrated dispensable to cell proliferation and apoptosis and it has been confirmed that inhibition of PI3K/Akt can help to suppress the proliferation of tumor cell [46]. Based on the results before, we can infer that IGFBP7-AS1 may regulate PI3K − Akt signaling pathway to alter the occurrence and development of tumors.

Notably, another essential finding in this study was the crucial role of IGFBP7-AS1 in immune response. TME plays a key role in tumor cell sustained growth, invasion and metastasis, and stromal cells and immune cells within TME represent attractive therapeutic targets with decreased risk of resistance and tumor recurrence [33]. Our study demonstrated that higher the expression of IGFBP7-AS1 and IGFBP7, the content of stromal and immune cells was higher, and the tumor purity was lower, which indicated that patient with higher expression level of IGFBP7-AS1 and IGFBP7 may have better therapeutic effect from immunotherapy.

When it comes to the correlation with immune related cells, IGFBP7-AS1 expression was negatively related with score of Macrophages M1 and M2, in accordance with the results from fraction variance of Macrophages M1 and M2 in different IGFBP7-AS1 groups. Many studies have shown on the one hand macrophage abundance in a tumor had an inverse correlation with patient survival, on the other hand it had a positive linkage with the resistance to chemotherapy [18]. Then, the increase in IGFBP7-AS1 and IGFBP7 expression indicated a higher presence of resting Mast cells. Mast cells could induce a privileged immunological environment with proliferation and the invasive ability of the cancer cells, as well as favoring the growth of a tumoral tissue with the development of its related vascular stroma (Aller, Arias, Arias, & Arias, [2]). Given that the significant infiltration of immune cells like macrophages, microglia, neutrophils, and monocytes may result in unfavorable prognosis in a number of cancer types [3], high IGFBP7-AS1 expression may indicate better UCEC overall survival. Also, high expressed IGFBP7-AS1 was accompanied with high score of TILs and immune-related substances like CCR, iDCs and HLA. These findings verified the prognostic ability of IGFBP7-AS1 in UCEC.

In recent years, significant advance has been made in studies focusing on T cells and related immune checkpoint, for their essential roles in immune infiltration and therapy. Blockade of inhibitory immune checkpoints can promote the recovery of T cell activation and prevent immune escape of cancer cells within the tumor microenvironment, while activation of stimulatory immune checkpoints can augment the effect of immune response (Y. [49]. To data, drugs that block immune checkpoints like PD1-PDL1 or CTLA4 have gradually become the mainstream of oncology. In our study, IGFBP7-AS1 and IGFBP7 expression were observed significant connected with various types of T cell, involving CD8 + T cells, T helper cells, T cell follicular helper, T cells gamma delta, Tregs, Th1 cells, and multiple immune checkpoints including PD1 and CTLA4 we mentioned before, suggesting that targeting IGFBP7-AS1 according to its traits in immune regulation of UCEC deserved research focus.

As previous studies demonstrated, several cancer types with high TMB may be attractive aims for immuno-oncology treatment development [10]. So, the results that high levels of IGFBP7-AS1 and IGFBP7 were both significantly associated with low TMB expression may provide implications for guidance on related immune therapy.

Finally, we also conducted pan-cancer analysis for verification, and drew the similar conclusion in most cancers. IGFBP7-AS1 expression was significantly associated with TMB, MSI, TME and Immune infiltration cells in pan-cancers, and impacted on the survival outcomes. Specifically, correlation between IGFBP7-AS1’s high expression and the upregulation of immune subtype C5 in pan-cancers was found. In previous study, IDH mutations has been reported enriched in C5, which associated with TME composition and decreased leukocyte chemotaxis, contributing to fewer tumor-associated immune cells and better clinical effect [42]. In drug sensitivity analysis, chemotherapeutic drug sensitivity of Veliparib, Mocetinostat, CYC-116 and Alisertib was negatively correlated with IGFBP7-AS1 expression, which may illustrate IGFBP7-AS1’s potential connection with their targets like PARP, HDAC and so on.

Besides, as the target gene of IGFBP7-AS1, IGFBP7 has been confirmed in previous study that inhibits IGF signaling by the way of binding the IGF-1 receptor (IGF-1R). IGF signaling activation is one of the major oncogenic events in diverse cancers, so IGFBP7 has been reported as a candidate tumor suppressor in several cancers [1],Y. [26, 35]. Moreover, IGFBP7 is manifested able to alter cell sensitivity to chemotherapeutic drugs, suggesting its beneficial value in anticancer therapies [43]. Considering the results from our study, we suggested IGFBP7-AS1 may be likely to influence on IGFBP7 expression, thereby affecting the UCEC patient’s survival.

The disadvantage of this study is that the molecule function of IGFBP7-AS1 in UCEC has still been uncovered and the results need further clinical verification. Therefore, a specific clinical cohort is needed to further assess the diagnostic and prognostic potential of IGFBP7-AS1 in UCEC. Moreover, we demonstrated that IGFBP7-AS1 has roles in multiple kinds of cancer, including LUAD, STAD and UCEC, and the emerging roles of IGFBP7-AS1 in these cancers are also waiting to be characterized.

## Conclusion

Taking together, increased IGFBP7-AS1 expression was related to positive prognosis of UCEC. Our comprehensive omics analysis revealed IGFBP7-AS1 may improve the tumor immune microenvironment, and impact on immune lymphocytes like T cells, Mast cells, Macrophages M1 and M2, including key immune checkpoints with its target gene IGFBP7, so that help to induce protective immunity in UCEC and provide a new reference for potential UCEC immunotherapy.

## Supplementary Information

**Additional file 1: Table S1.** Multivariate analysis result for the stage, grade and IGFBP7-AS1 expression only with OS of UCEC patients.

**Additional file 2: Figure S1.** Expression levels of IGFBP7-AS1 and IGFBP7 and association with overall survival in pan-cancer. (A, B) The expression of IGFBP7-AS1 and IGFBP7 in tumor and normal tissues across 33 pan-cancers. (C) The K-M survival curves of IGFBP7-AS1 expression groups in LAML, (D) LUAD, (E) MESO, (F) LGG, (G) STAD and (H) UCEC. (I) The forest plot for overall survival with 95% confidence intervals for 33 different cancer types.

**Additional file 3: Figure S2.** The relationship between gene expression and drug sensitivity.

## Data Availability

The data and materials used to support the findings of this study are available from the corresponding author on reasonable request.
