# Supplementary material for: A comprehensive prognostic and immune analysis of enhancer RNA identifies IGFBP7-AS1 as a novel prognostic biomarker in Uterine Corpus Endometrial Carcinoma
Source: Biol Proced Online. 2022 Jul 15;24:9. doi: 10.1186/s12575-022-00172-0 (PMC9284715; doi:10.1186/s12575-022-00172-0)
Supplement: Supplementary file 1 — Additional file 1: Table S1. Multivariate analysis result for the stage, grade and IGFBP7-AS1 expression only with OS of UCEC patients. [file 12575_2022_172_MOESM1_ESM.docx]

**Supplementary Table 1.** Multivariate analysis result for the stage, grade and IGFBP7-AS1 expression only with OS of UCEC patients.

| **Parameter** | **Multivariate analysis** | | | |
| --- | --- | --- | --- | --- |
|  | **HR** | **HR.95L** | **HR.95H** | **pvalue** |
| stage | 3.692 | 2.398 | 5.684 | 2.98E-09 |
| grade | 2.432 | 1.399 | 4.227 | 0.002 |
| IGFBP7-AS1 | 0.263 | 0.105 | 0.661 | 0.004 |
