# Supplementary figures and images for: A comprehensive prognostic and immune analysis of enhancer RNA identifies IGFBP7-AS1 as a novel prognostic biomarker in Uterine Corpus Endometrial Carcinoma
Source: Biol Proced Online. 2022 Jul 15;24:9. doi: 10.1186/s12575-022-00172-0 (PMC9284715; doi:10.1186/s12575-022-00172-0)

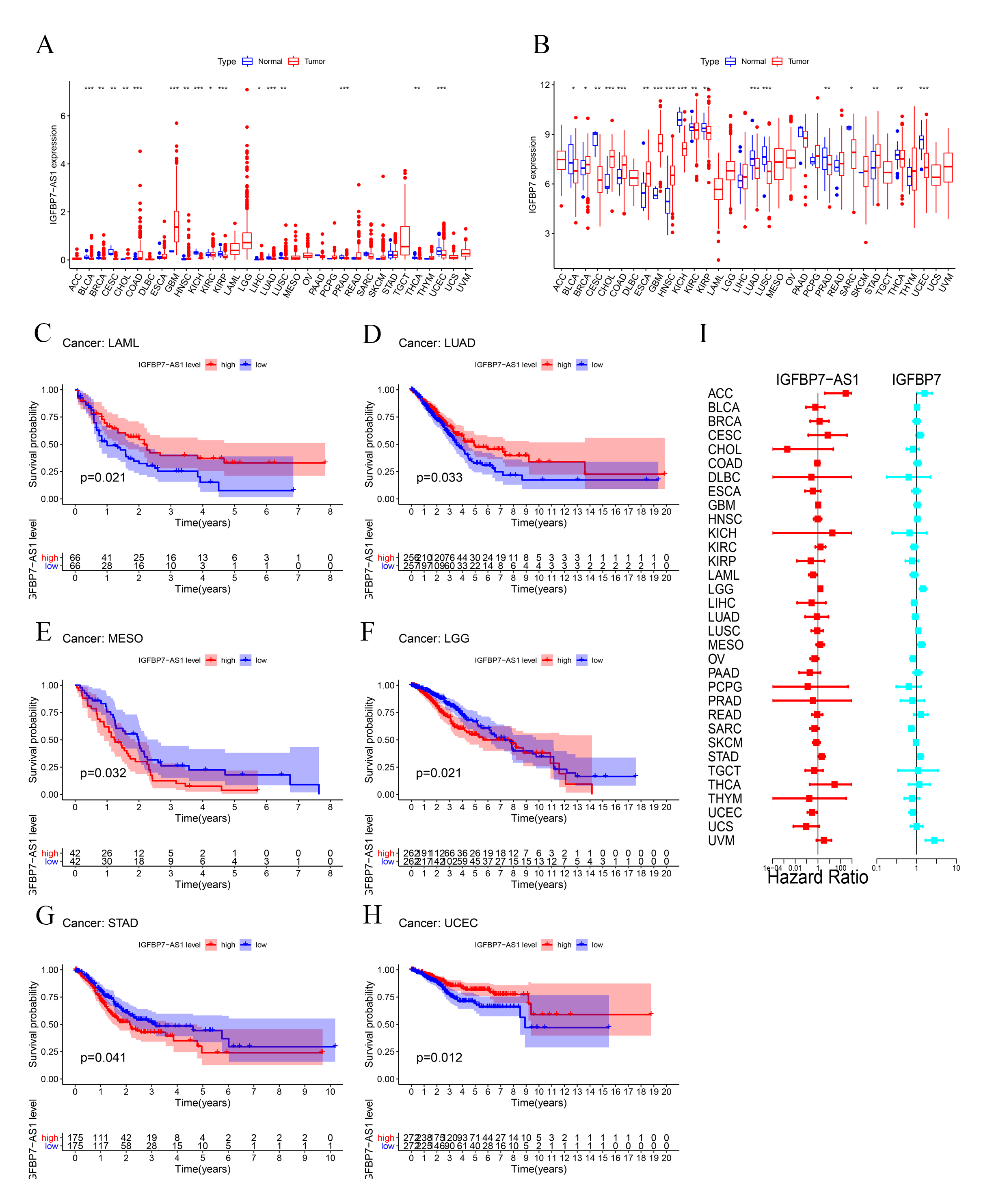

Supplement: Supplementary file 2 — Additional file 2: Figure S1. Expression levels of IGFBP7-AS1 and IGFBP7 and association with overall survival in pan-cancer. (A, B) The expression of IGFBP7-AS1 and IGFBP7 in tumor and normal tissues across 33 pan-cancers. (C) The K-M survival curves of IGFBP7-AS1 expression groups in LAML, (D) LUAD, (E) MESO, (F) LGG, (G) STAD and (H) UCEC. (I) The forest plot for overall survival with 95% confidence intervals for 33 different cancer types. [file 12575_2022_172_MOESM2_ESM.tif]

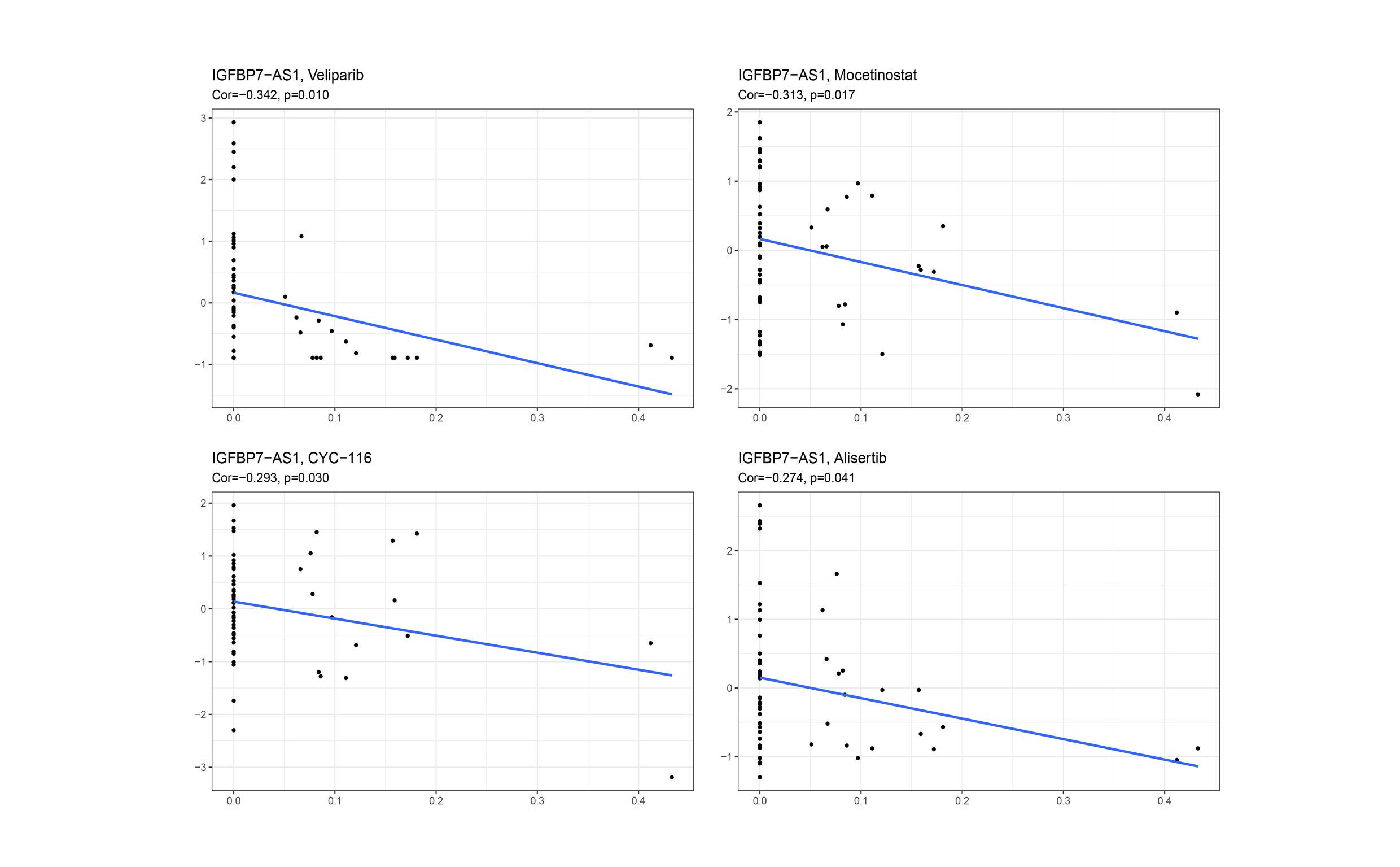

Supplement: Supplementary file 3 — Additional file 3: Figure S2. The relationship between gene expression and drug sensitivity. [file 12575_2022_172_MOESM3_ESM.tif]
